# Mathematical modelling reveals properties of TcdC required for it to be a negative regulator of toxin production in ***Clostridium difficile***

**DOI:** 10.1007/s00285-014-0780-0

**Published:** 2014-04-01

**Authors:** Sara Jabbari, Stephen T. Cartman, John R. King

**Affiliations:** 1 School of Mathematics and Institute of Microbiology and Infection, University of Birmingham, Birmingham, B15 2TT UK; 2 Clostridia Research Group, Centre for Biomolecular Sciences, University of Nottingham, Nottingham, NG7 2RD UK; 3 School of Mathematical Sciences, University of Nottingham, Nottingham, NG7 2RD UK

**Keywords:** Asymptotic analysis, *Clostridium difficile*, Gene regulation networks, Mathematical modelling, TcdC, 34E13, 92B05, 37N25

## Abstract

The role of the protein TcdC in pathogenicity of the bacterium *Clostridium difficile* is currently unclear: conflicting reports suggest it is either a negative regulator of toxin production or, on the other hand, has no effect on virulence at all. We exploit a theoretical approach by taking what is known about the network of proteins surrounding toxin production by *C. difficile* and translating this into a mathematical model. From there it is possible to investigate a range of possible interactions (using numerical and asymptotic analyses), identifying properties of TcdC which would make it a realistic candidate as a toxin inhibitor. Our findings imply that if TcdC is really an inhibitor of toxin production then TcdC production should be at least as fast as that of the protein TcdR and TcdC should remain in the cells throughout growth. These are experimentally-testable hypotheses and are equally applicable to alternative candidates for toxin production inhibition.

## Introduction

### *Clostridium difficile* and its pathogenicity

*Clostridium difficile* is a leading cause of hospital-associated infections in Europe, the United States and beyond. Infection generally occurs as a result of antibiotic use by a patient for a preceding infection: treatment causes disruption of the intestinal microflora, allowing colonisation by *C. difficile* in the gut (Rupnik et al. 2009). This Gram-positive organism has a range of effects on the host, from mild diarrhoea to pseudomembranous colitis (severe inflammation in areas of the colon) and thousands of cases every year in the United Kingdom alone are ultimately fatal (Statistical Bulletin 2011). Additionally, in surviving patients, infections often subsequently recur for months or years, even when treated with antibiotics. Worryingly, community outbreaks have begun to arise: *C. difficile* infection is no longer restricted to health care settings and a rise in what some believe are hypervirulent strains has been noted (Rupnik et al. 2009). Furthermore, antibiotic resistant strains are increasingly prevalent (French 2010). Evidently, as is the case with a growing number of bacterial species, novel drugs must be sought urgently.

One crucial obstacle to the development of targeted anti-*C. difficile* treatments is a general lack of knowledge about the virulence mechanisms used by this bacterium. Our understanding of the gene regulation networks governing pathogenesis in *Clostridium* species falls somewhat behind that of similar *Bacillus* species due to historical difficulties in genetically manipulating this bacterium. However, as more clostridial manipulation techniques are developed (Heap et al. 2007, 2010; O’Connor et al. 2006), one may expect to see an increase in the level of detail uncovered.

### The PaLoc

**Fig. 1:**
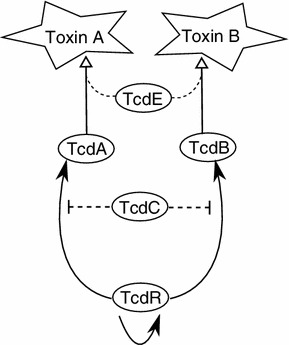
A representation of the PaLoc proteins and their mutual interactions. Disputed mechanisms are displayed with *dashed lines*. *White arrow heads* illustrate secretion and *black* ones transcription induction. Extracellular toxins are given in the *star* shapes, while intracellular proteins are *ovals*

In this study, we focus our attention on the subset of the overall network controlling *C. difficile* virulence that is most directly responsible for determining toxin production: the Pathogenicity Locus (PaLoc) comprising the five genes *tcdA, tcdB, tcdC, tcdE* and *tcdR* (see Fig. 1 and Hundsberger et al. 1997, for example).

The gene products TcdA and TcdB are the precursors for Toxins A and B respectively, the two principal toxins involved in *C. difficile* infection (Lyras et al. 2009; Kuehne et al. 2010; Carter et al. 2010).^1^ Transcription of *tcdA* and *tcdB* is positively controlled by TcdR, which also auto-regulates (Mani and Dupuy 2001; Mani et al. 2002). Conflicting reports exist surrounding the role of TcdE: it may (Tan et al. 2001; Govind and Dupuy 2012) or may not (Olling et al. 2012) be required for secretion of the toxins into the extracellular environment. Similarly, as we discuss below, recent publications have poured controversy on the role of TcdC.

### The role of TcdC

Before precise gene manipulation techniques were available for *C. difficile*, studies examining the role of TcdC had to be performed in a surrogate host, namely *C. perfringens*. Such work generated the hypothesis that TcdC negatively regulates toxin production via interference with the positive action of TcdR upon transcription of *tcdA* and *tcdB* (Dupuy et al. 2008; Matamouros et al. 2007). This was further supported with the discovery that (‘hypervirulent’) strains of *C. difficile*, believed to produce significantly higher toxin levels than previously seen strains, had a naturally occurring mutation in their *tcdC* gene, suggesting that these strains contain a non-functional TcdC protein that cannot temper toxin levels (Carter et al. 2011). However, two more recent studies in *C. difficile* itself (Cartman et al. 2012; Bakker et al. 2012) found that TcdC did not affect toxin levels in either the standard or ‘hypervirulent’ strains, drawing into question the role TcdC may have in regulating the amount of Toxin A and Toxin B produced. In addition, a mechanism of interaction between TcdC and TcdR has proved tricky to identify (van Leeuwen et al. 2013). Thus the role of TcdC, which could be crucial in *C. difficile* infection and in finding novel drug targets, remains unclear.

Mathematical modelling is increasingly being used as a means by which to glean information from gene regulation networks. Translating what is known about the system into a set of mathematical equations, and investigating various alternative assumptions about the more ambiguous elements, allows predictions to be made about the role of particular genes or proteins in the network and the effects of altering the expression levels of these genes and post-translational modifications to the system. We exploit this approach here to investigate the role of TcdC in the PaLoc and hence on toxin production by *C. difficile*.

## Model development

A standard procedure to build deterministic ordinary-differential-equation models of gene regulation networks in either prokaryotic or eukaryotic systems is to employ conventional mass-action kinetic theory (Karlebach and Shamir 2008, for example): the rate of a reaction is proportional to the product of the concentrations involved in the reaction. Variable and parameter definitions are provided in Tables 1 and 2. The following information and assumptions are used here to build (and in some cases simplify) a model of the PaLoc.

- The cells follow logistic growth (at rate $r$ with carrying capacity $K$) meaning that after an initial burst of exponential growth, the bacteria will settle into a stationary phase. This is appropriate for cells grown in batch culture over our time period of interest (roughly 24 h say).
- We assume TcdE is responsible for toxin secretion causing cell death in the process as per Govind and Dupuy (2012). Toxins are released following Michaelis–Menten kinetics at maximum rate $\mu$ with Michaelis constant $\eta$.
  1 $$\begin{aligned} \dfrac{dN}{dt}&= rN \left( 1-\dfrac{N}{K}\right) -\mu \left( \dfrac{E}{E+\eta }\right) N, \end{aligned}$$
  2 $$\begin{aligned} \dfrac{dX}{dt}&= \mu {A}\left( \dfrac{E}{E+\eta }\right) N. \end{aligned}$$
- Protein levels are proportional to their respective mRNA levels in terms of their dynamics (evidence suggests that this is the case at least for TcdA, TcdB and TcdC—Dupuy and Sonenshein (1998), Govind et al. (2006)). This allows us to include mRNA in the model implicitly, thus reducing the number of equations.
- Since we focus on qualitative implications, we consider only TcdA for simplicity (rather than both TcdA and TcdB), since they are believed to have the same dynamics but possibly at different quantitative levels (Dupuy and Sonenshein 1998).
- TcdA and TcdR are produced initially at a basal level during early growth (at rates $c_A^l$ and $c_R^l$ respectively) and at a higher rate induced by TcdR ($c_A^h$ and $c_R^h$) when TcdR is present in sufficient levels later in growth (Dupuy and Sonenshein 1998; Mani et al. 2002). TcdE, on the other hand, is produced basally at rate $c_E$ throughout, while *tcdC* transcription appears during exponential growth and is suppressed upon entry to stationary phase (Hundsberger et al. 1997; Govind et al. 2006; Carter et al. 2011). Indeed the last of these cited studies indicates that TcdC is present in the cells for the first 12 h of growth; this corresponds to the onset of stationary phase. We mark the transition between exponential and stationary phase (i.e. as the number of cells begins to settle off to the carrying capacity $K$), at time $t^*$, with a step function $F$:
  3 $$\begin{aligned} F=\left\{ \begin{array}{l@{\quad }l} 1&{} \hbox {if} \quad t<t^*\\ 0&{} \hbox {otherwise,} \end{array}\right. \end{aligned}$$
  but investigate also the possibility that TcdC is produced throughout growth.
- All proteins are subject to natural degradation (at rate $\lambda _x$ for variable $x$) and dilution as a result of cell division during growth.
  4 $$\begin{aligned} \dfrac{dE}{dt}&= c_E-\lambda _{E}E-rE\left( 1-\dfrac{N}{K}\right) , \end{aligned}$$
  5 $$\begin{aligned} \dfrac{dA}{dt}&= c_A^l+\dfrac{c_A^hR}{R+U_A/B_A}-\lambda _{A}A-rA \left( 1-\dfrac{N}{K}\right) . \end{aligned}$$
- If TcdC does indeed interfere with TcdR dynamics, the mechanism by which this would occur is unclear. In Carter et al. (2011), evidence suggests that TcdC operates at the transcriptional level, whereas van Leeuwen et al. (2013) indicate that TcdC is actually unlikely to bind the *tcdR* gene. Unless otherwise stated, we assume a functional TcdC protein inhibits toxin production by forming a heterodimer with TcdR (at rate $\beta$) thus blocking the positive action of TcdR on its own gene and on the toxin genes. This process whereby a protein is inactivated by heterodimerisation is known as molecular titration and is a common mechanism in regulatory networks to switch on or off a relevant response. See, for example, Buchler and Louis (2008) for a mathematical model demonstrating this. To model a strain with a non-functional TcdC protein, we simply use $\beta =0$. We assume binding is irreversible to examine functional TcdC at maximum efficiency. We include some simulations to illustrate that our conclusions apply also to the case where TcdC instead blocks *tcdR* transcription but limit this to numerical solutions for brevity.
  6 $$\begin{aligned} \dfrac{dR}{dt}&= c_R^l+\dfrac{c_R^hR}{R+U_R/B_R}-\beta {CR}-\lambda _{R}R-rR\left( 1-\dfrac{N}{K}\right) , \end{aligned}$$
  7 $$\begin{aligned} \dfrac{dC}{dt}&= c_CF-\beta {CR}-\lambda _{C}C-rC\left( 1-\dfrac{N}{K}\right) . \end{aligned}$$
  In the cases where TcdC acts at the transcriptional level, (6) and (7) are replaced respectively by
  8 $$\begin{aligned} \dfrac{dR}{dt}&= c_R^l+\dfrac{c_R^h(U_C/B_C)R}{(R+U_R/B_R)(C+U_C/B_C)}-\lambda _{R}R-rR\left( 1-\dfrac{N}{K}\right) , \end{aligned}$$
  9 $$\begin{aligned} \dfrac{dC}{dt}&= c_CF-\lambda _{C}C-rC\left( 1-\dfrac{N}{K}\right) . \end{aligned}$$

**Table 1:** Definitions of the model variables and their nondimensional scalings required to obtain Eqs. (10)–(17). In addition, time $(t)$ is scaled with $r$ (the rate of cell growth) to give the nondimensional $\tau =rt$

Variable	Concentration	Nondimensional form
$N$	Number of cells	${N}'=(1/K)N$
$E$	TcdE	${E}'=(r/c_E)E$
$A$	Intracellular TcdA	${A}'=(r/c_A^l)A$
$R$	TcdR	${R}'=(r/c_R^l)R$
$C$	TcdC	${C}'=(r/c_C)C$
$X$	Secreted toxin	${X}'=(r/Kc_A^l)X$

**Table 2:** Definitions of the parameters, their dimensions and their nondimensional representations in (10)–(17). We also include an estimate of the relative sizes of the nondimensional parameters

Parameter	Rate of	Dimensions	Nondimensional equivalent	Size
$r$	Cell growth	Time$^{-1}$	1	$O(1)$
$\mu$	Toxin secretion	Time$^{-1}$	$\mu '=\mu /r$	$O(\epsilon )$
$\eta$	Toxin secretion saturation constant	Concentration	$\eta '=\eta {r}/c_E$	$O(1)$
$c_A^l$	Constitutive TcdA production	Concentration time $^{-1}$	1	$O(1)$
$c_A^h$	TcdR-induced TcdA production	Concentration time $^{-1}$	$c=c_A^h/c_A^l$	$O(1/\epsilon )$
$c_E$	Constitutive TcdE production	Concentration time $^{-1}$	1	$O(1)$
$c_R^l$	Constitutive TcdR production	Concentration time $^{-1}$	1	$O(1)$
$c_R^h$	TcdR-induced TcdR production	Concentration time $^{-1}$	$c_R=c_R^h/c_R^l$	$O(1/\epsilon )$
$c_C$	Constitutive TcdC production	Concentration time $^{-1}$	1	$O(1)$
$\lambda _x$	Degradation of protein $x$	Time$^{-1}$	${\lambda '}_x=\lambda _x/r$	$O(1)$
$\lambda _C$	Degradation of TcdC	Time$^{-1}$	${\lambda '}_C=\lambda _C/r$	$O(\epsilon )$
$\beta$	Binding of TcdC and TcdR	Concentration$^{{-}1}$ time$^{-1}$	$\beta '=\beta {c_C}/{r^2}$	$O(1)$

Parameter	Rate constant for	Dimensions	Nondimensional equivalent	Size
$K$	Cell carrying capacity	Concentration	1	$O(1)$
$U_x/B_x$ ($x$ is $A$ or $R$)	Ratio of the separation to binding of TcdR from promoter site of *tcdx*	Concentration	$U_x'=U_xr/B_x{c_R^l}$	$O(1)$
$U_C/B_C$	Ratio of separation to binding of TcdC from promoter site of *tcdR*	Concentration	$U_C'=U_Cr/B_C{c_C}$	$O(1)$
–	Ratio of TcdC to basal TcdR production	–	$c_C^R=c_C/c_R^l$	$O(1/\epsilon )$

Given the lack of relevant quantitative data for the PaLoc, rather than attempting to obtain precise estimates for the parameters, we exploit asymptotic methods which require only the relative sizes of groups of parameters to be estimated. For this, we must nondimensionalise the system so that groupings are compared like for like (i.e. their dimensions are the same). Nondimensional scalings are listed in Tables 1 and 2 and the resulting nondimensional system becomes (dropping primes)

10 $$\begin{aligned} \dfrac{dN}{d\tau }&= N(1-N)-\epsilon \hat{\mu }\left( \dfrac{E}{E+\eta }\right) N, \end{aligned}$$

11 $$\begin{aligned} \dfrac{dE}{d\tau }&= 1-\lambda _{E}E-E(1-N), \end{aligned}$$

12 $$\begin{aligned} \dfrac{dA}{d\tau }&= 1+\dfrac{1}{\epsilon }\dfrac{\hat{c}R}{(R+U_A)}-\lambda _{A}A-A(1-N), \end{aligned}$$

13 $$\begin{aligned} \dfrac{dR}{d\tau }&= 1+\dfrac{1}{\epsilon }\dfrac{\hat{c}_RR}{(R+U_R)}-\beta {C}R-\lambda _{R}R-R(1-N), \end{aligned}$$

14 $$\begin{aligned} \dfrac{dC}{d\tau }&= F-\epsilon \dfrac{\beta }{\hat{c}_C^R}CR-\epsilon {\hat{\lambda }_{C}}C-C(1-N), \end{aligned}$$

15 $$\begin{aligned} \dfrac{dX}{d\tau }&= \epsilon \hat{\mu }{A}\left( \dfrac{E}{E+\eta }\right) N, \end{aligned}$$

with

16 $$\begin{aligned} \dfrac{ dR}{d\tau }&= 1+\dfrac{1}{\epsilon }\dfrac{\hat{c}_RU_CR}{(R+U_R)(C+U_C)}-\lambda _{R}R-R(1-N), \end{aligned}$$

17 $$\begin{aligned} \dfrac{dC}{d\tau }&= F-\epsilon {\hat{\lambda }_{C}}C-C(1-N) \end{aligned}$$

replacing (13) and (14) if TcdC inhibits TcdR production at the transcriptional level. We include, in the above, scalings of the nondimensional parameters (denoted by hats) according to a small parameter $\epsilon$ which arises naturally in the ratio of constitutive to TcdR-induced transcription levels, the first of which is naturally much smaller than the second. The scalings for $c=c_A^h/c_A^l$ and $c_R=c_R^h/c_R^l$ follow. To increase the possibility that TcdC has an effect upon the system dynamics, we choose also to use $c_C^R=O(1/\epsilon )$ and $\lambda _{C}=O(\epsilon )$. Finally, since we assume cells die through lysis when toxins are secreted, we take the rate of toxin secretion, $\mu$, to be relatively slow to avoid the population dying out unrealistically quickly.

## Results

### Intracellular dynamics

We solve the nonlinear system (10)–(15) numerically (using ode23 in Matlab) to yield a first level of insight into the dynamics of the components of the PaLoc. Since the time taken for the cells to enter stationary phase in our simulations is roughly $\tau =10$, we choose this as our cut-off point for TcdC transcription, i.e.

18 $$\begin{aligned} F=\left\{ \begin{array}{l@{\quad }l} 1&{} \hbox {if} \quad \tau <10\\ 0&{} \hbox {otherwise,} \end{array}\right. \end{aligned}$$

(though using a step function generates a rather sharp transition in TcdC levels, the qualitative conclusions are not affected by adopting a smoother function to represent this switch).

**Fig. 2:**
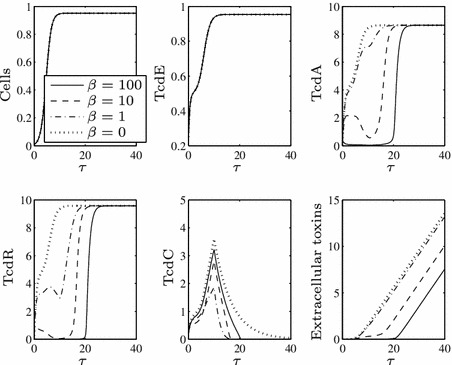
Numerical solutions to (10)–(15) using $\epsilon =0.1$ (i.e. small, as required) and a range of values of $\beta$, representing the rate of TcdC binding to TcdR. Large values of $\beta$ lead to lowered TcdA and toxin levels while TcdC is present in the cells, but they ultimately cannot prevent toxin levels attaining those seen for $\beta =0$, albeit at a later time point. Here, and henceforth, we use the arbitrary initial conditions $N(0)=0.01$, $X(0)=0$, $A(0)=E(0)=R(0)=C(0)=0.25$ representing the cells not producing substantial levels of toxins initially

In order to investigate the role of TcdC in toxin production we consider various values of $\beta$ (the rate of TcdR inactivation by TcdC), see Fig. 2. We find

- for sufficiently large values of $\beta$, toxin production can be lowered in the presence of functional TcdC, but as soon as TcdC disappears from the cells through natural degradation (around $\tau \approx {20}$), toxin levels are restored to the level achieved if TcdC cannot bind TcdR (compare to the dotted line of Fig. 2). The strength of TcdR to activate toxin production in this phase is further increased by the fact it is positively auto-regulated, meaning that by the later stages of growth TcdR levels increase significantly.
- When toxin levels are lowered (in $\tau <20$), they reach extremely low levels (consistent with the ultra-sensitive nature of switches that can be induced by molecular titration—Buchler and Louis 2008) which may not be realistic—the non-hypervirulent strains of *C. difficile* involved in infection are indeed still virulent, implying that at least some toxins are present, as is the case in all studies of TcdC (regardless of the findings regarding its role).
- For all positive values of $\beta$, toxin production eventually reaches the levels seen for $\beta =0$ (i.e. the ‘hypervirulent’ case, dotted line), merely at a later time point.
- For lower values of $\beta$ (see the dot-dash line), toxin production is barely reduced even in early growth and very quickly attains ‘hypervirulent’ levels.

**Fig. 3:**
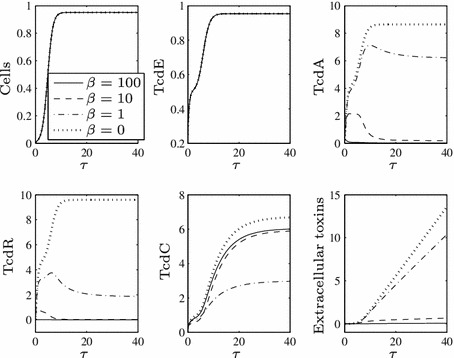
Numerical solution to (10)–(15) when $F=1$ for all $\tau$, $\epsilon =0.1$ and varying $\beta$ values. When TcdC is produced throughout growth, it can have a long-term negative effect on toxin levels, even for low values of $\beta$

Figure 3 enables us to visualise what would arise if TcdC production were to occur constitutively throughout cell growth (i.e. if $F=1$ for all $\tau$).

- As TcdC approaches its non-zero steady state, TcdR proteins are impeded, preventing a long-term increase in TcdA and subsequently toxin levels.
- Setting $\beta =0$ and removing this TcdC-induced obstruction (dotted line) induces significantly higher toxin levels, both during the transient dynamics and in the steady state of TcdA (notice that toxin levels even for $\beta =1$, given by the dot-dash line, are markedly lower than those for $\beta =0$).

**Fig. 4:**
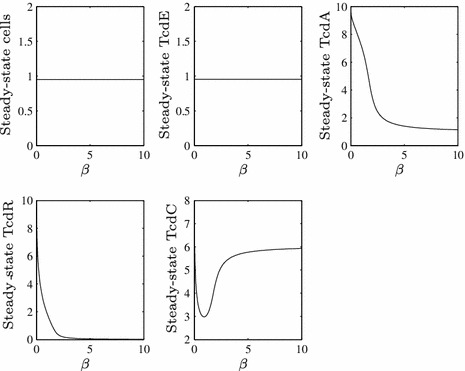
A steady-state plot of the system in response to changes in $\beta$ $(\epsilon =0.1)$ if TcdC is produced throughout growth ($F=1$ for all $\tau$). We omit extracellular toxin levels from these solutions since they never achieve steady state under our assumptions (as we assume toxins are secreted as long as cells and TcdA are present), but any effect on toxin levels can be inferred from the solution for TcdA (since extracellular toxins are downstream of the rest of the system, this has no consequence on any other variable). Notice that the dip in TcdC levels occurs because TcdC is free (unbound) at low $\beta$, while for large $\beta$ TcdR is sequestered sufficiently early for TcdC to be produced and remain free in the cell with no additional TcdR to bind. Intermediate values of $\beta$ result in a decrease in TcdC as a result of binding to TcdR. Given that the system is monostable, our simple choice of initial conditions given in Fig. 2 does not affect the steady state of the system

The fact that low toxin production cannot be maintained once TcdC vanishes from the cells suggests that the system is monostable in our parameter range and Fig. 4 confirms this for varying $\beta$ (this steady-state plot was created using the bifurcation and continuation software XPPAUT).

We observe that if TcdC is produced constitutively throughout growth and $\beta$ is greater than roughly 3 (for $\epsilon =0.1$) then TcdC has a significant negative effect on TcdA (and consequently on toxins). However, as alluded to earlier, if $\beta$ is sufficiently large, it is possible that it actually reduces TcdA to unrealistically low levels, leaving only a small interval of $\beta$ that would yield results that might be observed in practice. We shall see in Sect. 3.3, where we shift our focus to the parameter $\hat{c}_C^R$, that altering production rather than binding rates could produce more realistic levels.

These numerical results suggest that for non-functional TcdC to be the cause of any hypervirulence in *C. difficile*, it should be produced throughout growth. If TcdC is produced only in early growth then $\beta$ must be sufficiently large for it to have any effect in the short to medium term, but eventually maximum toxin levels will be achieved once TcdC disappears from the cells, preventing functional TcdC from being the cause of lower toxin levels in the long term.

**Fig. 5:**
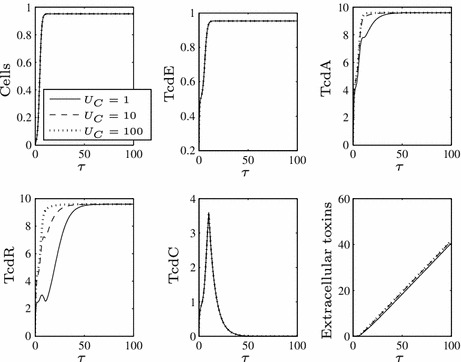
Time-dependent numerical solution to the full system, assuming TcdC inhibits TcdR production at the transcriptional level (Eqs. (10)–(12) and (15)–(17) with $\epsilon =0.1$). The *solid line* represents full inhibition $(U_C=1)$, while the *dashed* $(U_C=O(1/\epsilon ))$ and *dotted* $(U_C=O(1/\epsilon ^2))$ *lines* illustrate an increasing inability for TcdC to bind and block *tcdR* transcription (i.e. this represents the hypervirulent case). Toxin production is only mildly suppressed while TcdC is present in the cells

**Fig. 6:**
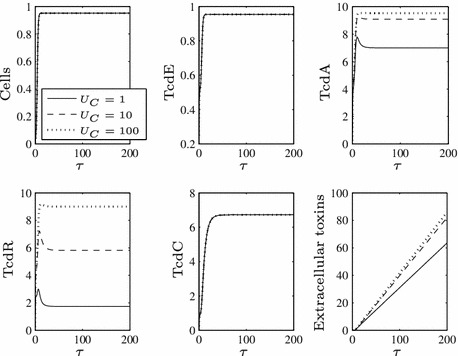
Time-dependent numerical solution to the full system, assuming TcdC inhibits TcdR production at the transcriptional level and is produced throughout growth (Eqs. (10)–(12) and (15)–(17) with $\epsilon =0.1$ and $F=1$ for all $\tau$). The *solid line* represents full inhibition $(U_C=1)$, while the *dashed* $(U_C=O(1/\epsilon ))$ and *dotted* $(U_C=O(1/\epsilon ^2))$ *lines* illustrate an increasing inability for TcdC to bind and block *tcdR* transcription (i.e. this represents the hypervirulent case). As with the case where TcdC binds TcdR, in this scenario toxin production *can* be lowered in the long term

In all these simulations, we have assumed the mode of inhibition by TcdC is through binding to TcdR. In Figs. 5 and 6 we illustrate that equivalent conclusions hold if TcdC instead blocks *tcdR* transcription, though in this case toxin levels never reach amounts as low as in the previous case.

In the following sections we exploit asymptotic analyses to uncover the timescale on which any effect on toxin production by TcdC could first manifest itself (Sect. 3.2) and how fast TcdC must be produced (in relation to the other proteins in the PaLoc) for it to have a long-term effect on toxin levels (Sect. 3.3). In the interests of brevity, we limit this to the model representing TcdC-TcdR heterodimerisation but the methods applied therein are equally applicable to the model whereby TcdC acts via transcription.

### Time-dependent asymptotic analysis

In this section we split the full transient solution into distinct timescales where different reactions dominate in each case (see Jabbari et al. 2010 for an additional example of such time-dependent asymptotic analysis in the context of gene regulation networks). This enables mathematical visualisation of when TcdC first has an effect on toxin levels, providing an estimate for the minimum time TcdC must exist in the cells for it to exert its hypothesised effect. In addition, the simple analytical expressions will facilitate parameter inference from time course measurements of protein levels or gene expression in future work. Three timescales emerge from the asymptotic analysis; the final two must be split into three cases depending on the value of what we will show to be a key combination of parameters: $F\hat{c}_C^R-\hat{c}_R$ (in fact when this is negative the system resolves to steady state already on the second timescale). We summarise the analysis in Table 3 and include full details in the Appendix.

**Table 3:** A summary of the scalings required for the time-dependent asymptotic analysis of Sect. 3.2 and the large time behaviour of each variable on each timescale (since this facilitates derivation of the scalings for the subsequent timescale). The final two timescales differ depending on the value of $F\hat{c}_C^R-\hat{c}_R$. When $F\hat{c}_C^R-\hat{c}_R<0$, the system settles to steady state already on the second timescale. We have that $\rho =\hat{\lambda }_C+\hat{\mu }/(1+\eta \lambda _E)-FU_R\beta /\hat{c}_R$ and $\theta =F\lambda _R/\beta$. We replace the steady state approximations for $A$, $R$ and $C$ by the relevant equation number for the final timescale of Case III in the interests of space

Timescale	$F\hat{c}_C^R-\hat{c}_R$	Scalings						Large $\tau$ behaviour				
		$\tau$	$N$	$E$	$A$	$R$	$C$	$N$	$E$	$A$	$R$	$C$
1	–	$\epsilon \hat{\tau }$	–	–	–	–	–	$N(0)$	$E(0)$	$\hat{c}\hat{\tau }$	$\hat{c}_R\hat{\tau }$	$C(0)$
2, Case I	$<$0	$\tilde{\tau }$	–	–	$\epsilon ^{-1}\tilde{A}$	$\epsilon ^{-1}\tilde{R}$	–	${1}$	$\frac{1}{\lambda _E}$	$\frac{\hat{c}}{\lambda _A}$	$\frac{\hat{c}_R-F\hat{c}_C^R}{\lambda _R}$	$\frac{F\hat{c}_C^R\lambda _R}{\beta (\hat{c}_R-F\hat{c}_C^R)}$
2, Case II	$=$0	$\tilde{\tau }$	–	–	$\epsilon ^{-1}\tilde{A}$	$\epsilon ^{-1}\tilde{R}$	–	${1}$	$\frac{1}{\lambda _E}$	$\frac{\hat{c}}{\lambda _A}$	$\frac{F\hat{c}_C^R}{\beta }\left( \frac{2F\lambda _R}{\beta }\tilde{\tau }\right) ^{-\frac{1}{2}}$	$\left( \frac{2F\lambda _R}{\beta }\tilde{\tau }\right) ^{\frac{1}{2}}$
2, Case III	$>$0	$\tilde{\tau }$	–	–	$\epsilon ^{-1}\tilde{A}$	$\epsilon ^{-1}\tilde{R}$	–	${1}$	$\frac{1}{\lambda _E}$	$\frac{\hat{c}}{\lambda _A}$	$\frac{\hat{c}_R\hat{c}_C^R}{\beta (F\hat{c}_C^R-\hat{c}_R)\tilde{\tau }}$	$\left( \frac{F\hat{c}_C^R-\hat{c}_R}{\hat{c}_C^R}\right) \tilde{\tau }$
3, Case II	$=$0	$\epsilon ^{-1}\check{\tau }$	–	–	$\epsilon ^{-1}\tilde{A}$	$\epsilon ^{-\frac{1}{2}}\check{R}$	$\epsilon ^{-\frac{1}{2}}\check{C}$	1	$\frac{1}{\lambda _E}$	$\frac{\hat{c}}{\lambda _A}$	$\frac{\hat{c}_R}{\beta }\sqrt{\frac{\rho }{\theta }}$	$\sqrt{\frac{\theta }{\rho }}$
3, Case III	$>$0	$\epsilon ^{-1}\check{\tau }$	–	–	$\epsilon ^{-1}\tilde{A}$	$\check{R}$	$\epsilon ^{-1}\check{C}$	1	$\frac{1}{\lambda _E}$	(61)	(60)	(59)

Given that secreted toxin levels never achieve steady state under our assumptions, for simplicity we drop this variable from the remainder of the study and infer all results from TcdA, the precursor to secreted toxins.

#### Timescale 1: $\tau =\epsilon \hat{\tau }$

On this first timescale, all variables except $\tau$ are $O(1)$. The system (10)–(14) becomes

19 $$\begin{aligned} \dfrac{dN}{d\hat{\tau }}&= \epsilon {N}(1-N)-\epsilon ^2\hat{\mu } \left( \dfrac{E}{E+\eta }\right) N, \end{aligned}$$

20 $$\begin{aligned} \dfrac{dE}{d\hat{\tau }}&= \epsilon -\epsilon \lambda _{E}E-\epsilon {E}(1-N), \end{aligned}$$

21 $$\begin{aligned} \dfrac{dA}{d\hat{\tau }}&= \epsilon +\dfrac{\hat{c}R}{R+U_A}-\epsilon \lambda _{A}A-\epsilon {A}(1-N), \end{aligned}$$

22 $$\begin{aligned} \dfrac{dR}{d\hat{\tau }}&= \epsilon +\dfrac{\hat{c}_RR}{R+U_R}-\epsilon \beta {C}R-\epsilon \lambda _{R}R-\epsilon {R}(1-N), \end{aligned}$$

23 $$\begin{aligned} \dfrac{dC}{d\hat{\tau }}&= \epsilon {F}-\epsilon ^2\hat{\lambda }_{C}C-\epsilon ^2\dfrac{\beta }{\hat{c}_C^R}CR-\epsilon {C}(1-N), \end{aligned}$$

meaning that the $O(1)$ (i.e. the largest) terms yield the following leading-order behaviour:

24 $$\begin{aligned}&N=N(0), \quad E=E(0), \quad C=C(0), \end{aligned}$$

25 $$\begin{aligned}&R+U_R\ln (R) = \hat{c}_R\hat{\tau }+R(0)+U_R\ln (R(0)), \end{aligned}$$

26 $$\begin{aligned}&A = \int \dfrac{\hat{c}R}{R+U_A}\,d\hat{\tau }. \end{aligned}$$

**Fig. 7:**
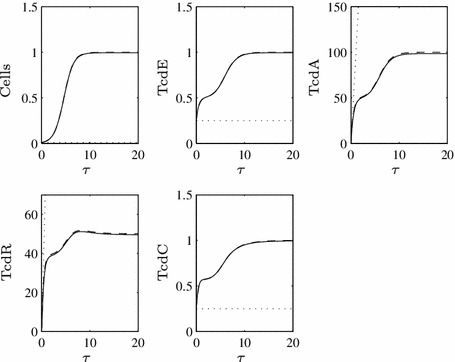
Time-dependent numerical solution to the full system (Eqs. (10)–(15), *solid line*), with the asymptotic approximations on the first (*dotted line*) and second (*dashed line*) timescales when $\hat{c}_C^R=0.5$, i.e. Case I, and $\epsilon =0.01$ (we use a smaller value of $\epsilon$ in this section to demonstrate better the close fit of the asymptotic approximations). Initial conditions on the first timescale are as stated for Fig. 2 while on the second timescale, the matching conditions $N(0)=0.01$, $E(0)=C(0)=0.25$, $A(0)=R(0)=0$ hold

**Fig. 8:**
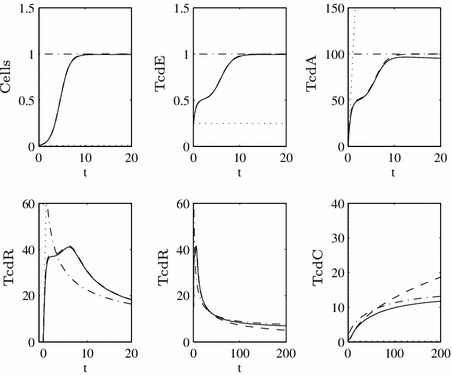
Time-dependent numerical solution to the full system (*solid line*), with the asymptotic approximations on the first (*dotted line*), second (*dashed line*) and third (*dot-dashed line*) timescales when $\hat{c}_C^R=1$, i.e. Case II, and $\epsilon =0.01$. We depict the approximations for TcdR twice to better illustrate how they match the full solution on distinct timescales

**Fig. 9:**
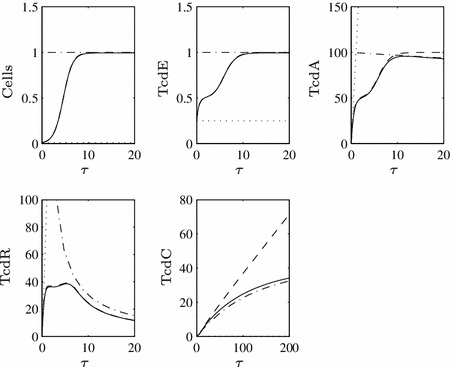
Time-dependent numerical solution to the full system (*solid line*), with the asymptotic approximations on the first (*dotted line*), second (*dashed line*) and third (*dot-dashed line*) timescales when $c_C^R=1.5$, i.e. Case III, and $\epsilon =0.01$

Only TcdA and TcdR dynamics evolve on this very early timescale, both seeing the positive influence of TcdR upon their production rates. Notably, TcdC remains at its initial value. These approximations to the behaviour on an early timescale are depicted in Figs. 7, 8, and 9 (division into parameter-led subcases on subsequent timescales requires multiple plots).

#### Timescale 2: $\hat{\tau }=\epsilon ^{-1}\tilde{\tau }$, $A=\epsilon ^{-1}\tilde{A}$, $R=\epsilon ^{-1}\tilde{R}$

The influence of TcdR upon TcdA and its own production results in $A$ and $R$ increasing on this timescale. The resulting rescaled problem reads

27 $$\begin{aligned} \dfrac{dN}{d\tilde{\tau }}&= N(1-N)-\epsilon \hat{\mu }\left( \dfrac{E}{E+\eta }\right) N, \end{aligned}$$

28 $$\begin{aligned} \dfrac{dE}{d\tilde{\tau }}&= 1-\lambda _{E}E-E(1-N), \end{aligned}$$

29 $$\begin{aligned} \dfrac{d\tilde{A}}{d\tilde{\tau }}&= \epsilon +\dfrac{\hat{c}\tilde{R}}{\tilde{R}+\epsilon {U_A}}-\lambda _{A}\tilde{A}-\tilde{A}(1-N), \end{aligned}$$

30 $$\begin{aligned} \dfrac{d\tilde{R}}{d\tilde{\tau }}&= \epsilon +\dfrac{\hat{c}_R\tilde{R}}{\tilde{R}+\epsilon {U_R}}-\beta {C}\tilde{R}-\lambda _{R}\tilde{R}-\tilde{R}(1-N), \end{aligned}$$

31 $$\begin{aligned} \dfrac{dC}{d\tilde{\tau }}&= F-\epsilon \hat{\lambda }_{C}C-\dfrac{\beta }{\hat{c}_C^R}C\tilde{R}-C(1-N). \end{aligned}$$

Notice that the equations for $N$, $E$ and $A$ decouple from the remaining system at leading order and can be solved to give

32 $$\begin{aligned} N&= \dfrac{N(0)e^{\tilde{\tau }}}{1+N(0)(e^{\tilde{\tau }}-1)}, \end{aligned}$$

33 $$\begin{aligned} E&= \left( \dfrac{1-N(0)}{e^{\tilde{\tau }(\lambda _{E}+1)}}+\dfrac{N(0)}{e^{\lambda _{E}\tilde{\tau }}}\right) \int \dfrac{e^{\tilde{\tau }(\lambda _{E}+1)}}{1-N(0)+N(0)e^{\tilde{\tau }}}\,d\tilde{\tau }, \end{aligned}$$

34 $$\begin{aligned} \tilde{A}&= \left( \dfrac{1-N(0)}{e^{\tilde{\tau }(\lambda _{A}+1)}}+\dfrac{N(0)}{e^{\lambda _{A}\tilde{\tau }}}\right) \int \dfrac{\hat{c}e^{\tilde{\tau }(\lambda _{A}+1)}}{1-N(0)+N(0)e^{\tilde{\tau }}}\,d\tilde{\tau }, \end{aligned}$$

where

35 $$\begin{aligned} N\sim {1}, \qquad E \sim \dfrac{1}{\lambda _{E}},\qquad \tilde{A}\sim \dfrac{\hat{c}}{\lambda _{A}} \end{aligned}$$

for large $\tilde{\tau }$. At leading order therefore, cell number is governed solely by the logistic equation, toxin production not being significant enough to negatively impact on growth.

Adopting (35) and combining the leading-order terms of (30) and (31) enables us to obtain the large $\tilde{\tau }$ behaviour of $C$ and $\tilde{R}$:

36 $$\begin{aligned} \dfrac{d}{d\tilde{\tau }}(\hat{c}_C^RC-\tilde{R}) \sim (F\hat{c}_C^R-\hat{c}_R)+\lambda _{R}\tilde{R}. \end{aligned}$$

The dynamics of these two variables are determined by whether $F\hat{c}_C^R-\hat{c}_R$ is negative, zero or positive. We examine each case separately (note that (35) holds for all three scenarios outlined below).

*Case I:* $$F\hat{c}_C^R< \hat{c}_R$$

If $F\hat{c}_C^R< \hat{c}_R$, the leading-order system evolves to steady state on this second timescale, namely

37 $$\begin{aligned} \bar{R}\sim \dfrac{\hat{c}_R-F\hat{c}_C^R}{\lambda _{R}}, \qquad \qquad \bar{C}\sim \dfrac{F\hat{c}_C^R\lambda _{R}}{\beta (\hat{c}_R-F\hat{c}_C^R)}, \end{aligned}$$

see Fig. 7. TcdA is not affected by any parameters relating to TcdC at leading order since TcdR is produced at a faster rate than TcdC, thus rendering TcdC ineffective. Instead, throughout the whole time course of the simulation TcdA is governed solely by its own production rate, balanced by its loss rate.

*Case II:* $$F\hat{c}_C^R=\hat{c}_R$$

Following the analysis detailed in the Appendix, we find

38 $$\begin{aligned} C\sim \left( \dfrac{2F\lambda _{R}}{\beta }\tilde{\tau }\right) ^{\frac{1}{2}} \end{aligned}$$

and

39 $$\begin{aligned} \tilde{R}\sim \dfrac{F\hat{c}_C^R}{\beta } \left( \dfrac{2F\lambda _{R}}{\beta }\tilde{\tau }\right) ^{-\frac{1}{2}}, \end{aligned}$$

for large $\tilde{\tau }$. In this instance, $\hat{c}_C^R$ is influencing TcdR levels on this intermediary timescale but the effect has not yet filtered through to TcdA.

*Case III:* $$F\hat{c}_C^R>\hat{c}_R$$

Under this parameter regime,

40 $$\begin{aligned} C\sim \left( \dfrac{F\hat{c}_C^R-\hat{c}_R}{\hat{c}_C^R}\right) \tilde{\tau } \end{aligned}$$

and

41 $$\begin{aligned} \tilde{R}\sim \dfrac{\hat{c}_R\hat{c}_C^R}{\beta (F\hat{c}_C^R-\hat{c}_R)\tilde{\tau }} \end{aligned}$$

hold for large $\tilde{\tau }$. As above, $\hat{c}_C^R$ is not yet affecting TcdA on this timescale, but it does have a significant effect on both TcdC and TcdR, with the difference between $\hat{c}_C^R$ and $\hat{c}_R$ being key to determining the rates at which these proteins are produced. In addition, in contrast to Case II where an increase in the rate of binding of TcdR and TcdC negatively impacts on the levels of both of these proteins, here (where TcdC production occurs faster than that of TcdR) $\beta$ affects only TcdR at leading order because TcdC is produced in sufficient quantities for it to be replenished following loss through complex formation. This will prove to be a key property of TcdC for it to be a negative regulator of toxin production in this study.

#### Timescale 3

*Case I:* $$F\hat{c}_C^R< \hat{c}_R$$

When $F\hat{c}_C^R< \hat{c}_R$, the system has already evolved to steady state on Timescale 2 and therefore no further discussion is needed here.

*Case II:* $$F\hat{c}_C^R=\hat{c}_R$$, $$\tilde{\tau }=\epsilon ^{-1}\check{\tau }$$, $$C=\epsilon ^{-\frac{1}{2}}\check{C}$$, $$\tilde{R}=\epsilon ^{\frac{1}{2}}\check{R}$$

On the third timescale, if the rates of TcdC and TcdR production are identical, we have

42 $$\begin{aligned} \epsilon \dfrac{dN}{d\check{\tau }}&= N(1-N)-\epsilon \hat{\mu }\left( \dfrac{E}{E+\eta }\right) N, \end{aligned}$$

43 $$\begin{aligned} \epsilon \dfrac{dE}{d\check{\tau }}&= 1-\lambda _{E}E-E(1-N), \end{aligned}$$

44 $$\begin{aligned} \epsilon \dfrac{d\tilde{A}}{d\check{\tau }}&= \epsilon +\dfrac{\hat{c}\check{R}}{\check{R}+\epsilon ^{\frac{1}{2}}U_A}-\lambda _{A}\tilde{A}-\tilde{A}(1-N), \end{aligned}$$

45 $$\begin{aligned} \epsilon ^{\frac{3}{2}}\dfrac{d\check{R}}{d\check{\tau }}&= \epsilon +\dfrac{\hat{c}_R\check{R}}{\check{R}+\epsilon ^{\frac{1}{2}}U_R}-\beta {\check{C}}{\check{R}}-\epsilon ^{\frac{1}{2}}\lambda _{R}\check{R}-\epsilon ^{\frac{1}{2}}{\check{R}}(1-N), \end{aligned}$$

46 $$\begin{aligned} \epsilon \dfrac{d\check{C}}{d\check{\tau }}&= \epsilon ^{\frac{1}{2}}F-\epsilon \hat{\lambda }_{C}\check{C}-\epsilon ^{\frac{1}{2}}\dfrac{\beta }{\hat{c}_C^R}\check{C}\check{R}-\check{C}(1-N). \end{aligned}$$

The first three of these equations give

47 $$\begin{aligned} N\sim 1-\epsilon \hat{\mu }\left( \dfrac{1}{1+\eta \lambda _{E}}\right) , \end{aligned}$$

(the $O(\epsilon )$ term being needed to obtain the leading-order balance in (46)),

48 $$\begin{aligned} E\sim \dfrac{1}{\lambda _{E}},\qquad \qquad \tilde{A}\sim \dfrac{\hat{c}}{\lambda _{A}}, \end{aligned}$$

meaning that, again, TcdA is *not* affected by TcdC at leading order at any point in the time course. We find (see Appendix)

49 $$\begin{aligned} \check{C}&\sim \dfrac{\sqrt{\rho \theta (1-e^{-2\rho \check{\tau }})}}{\rho }, \end{aligned}$$

50 $$\begin{aligned} \check{R}&\sim \dfrac{\hat{c}_R\rho \sqrt{\rho \theta (1-e^{-2\rho \check{\tau }})}}{\beta \rho \theta (1-e^{-2\rho \check{\tau }})}, \end{aligned}$$

where

51 $$\begin{aligned} \rho =\hat{\lambda }_C+\dfrac{\hat{\mu }}{1+\eta \lambda _E}-\dfrac{FU_R\beta }{\hat{c}_R} \quad \hbox { and } \quad \theta =\dfrac{F\lambda _R}{\beta }. \end{aligned}$$

The resulting approximations are depicted in Fig. 8.

Remember that in this scenario $F\hat{c}_C^R=\hat{c}_R$, which in terms of the dimensional parameters requires that the rate of TcdC production is exactly the same as the fastest rate of TcdR production. Though it is nigh impossible that this occur in reality, we include the analysis here for mathematical completeness. Given that the rate of protein production is unlikely to be absolutely constant, this could in theory cater for the scenario where the production rates of TcdC and TcdR waver around similar values.

*Case III:* $$F\hat{c}_C^R>\hat{c}_R$$, $$\tilde{\tau }=\epsilon ^{-1}\check{\tau }$$, $$C=\epsilon ^{-1}\check{C}$$, $$\tilde{R}=\epsilon \check{R}$$

In this case, where TcdC production occurs at a faster rate than that of TcdR, we have on the final timescale:

52 $$\begin{aligned} \epsilon \dfrac{dN}{d\check{\tau }}&= N(1-N)-\epsilon \hat{\mu }\left( \dfrac{E}{E+\eta }\right) N, \end{aligned}$$

53 $$\begin{aligned} \epsilon \dfrac{dE}{d\check{\tau }}&= 1-\lambda _{E}E-E(1-N), \end{aligned}$$

54 $$\begin{aligned} \epsilon \dfrac{d\tilde{A}}{d\check{\tau }}&= \epsilon +\dfrac{\hat{c}\check{R}}{\check{R}+U_A}-\lambda _{A}\tilde{A}-\tilde{A}(1-N), \end{aligned}$$

55 $$\begin{aligned} \epsilon ^2\dfrac{d\check{R}}{d\check{\tau }}&= \epsilon +\dfrac{\hat{c}_R\check{R}}{\check{R}+U_R}-\beta {\check{C}}{\check{R}}-\epsilon \lambda _{R}\check{R}-\epsilon {\check{R}}(1-N), \end{aligned}$$

56 $$\begin{aligned} \epsilon \dfrac{d\check{C}}{d\check{\tau }}&= \epsilon {F}-\epsilon \hat{\lambda }_{C}\check{C}-\epsilon \dfrac{\beta }{\hat{c}_C^R}\check{C}\check{R}-\check{C}(1-N), \end{aligned}$$

so that (47) holds also in this case. The remaining approximations are given by

57 $$\begin{aligned} E&\sim \dfrac{1}{\lambda _{E}}, \quad \check{R}\sim \dfrac{\hat{c}_R}{\beta {\check{C}}}-U_R,\quad \tilde{A}\sim \dfrac{\hat{c}}{\lambda _{A}}\left( \dfrac{\hat{c}_R-U_R\beta \check{C}}{\hat{c}_R+(U_A-U_R)\beta \check{C}}\right) , \end{aligned}$$

58 $$\begin{aligned} \check{C}&\sim \dfrac{(F\hat{c}_C^R-\hat{c}_R)(1+\eta \lambda _{E})}{(1+\eta \lambda _{E})(\beta {U_R}-\hat{\lambda }_C\hat{c}_C^R)-\hat{\mu }\hat{c}_C^R}\left( \exp ((\frac{\beta {U_R}}{\hat{c}_C^R}-\hat{\lambda }_{C}-\frac{\hat{\mu }}{1+\eta \lambda _{E}})\check{\tau })-1\right) ,\nonumber \\ \end{aligned}$$

The approximations for $\check{R}$ and $A$ follow.

At steady state therefore we have

59 $$\begin{aligned} \bar{\check{C}}&= \dfrac{(F\hat{c}_C^R-\hat{c}_R)(1+\eta \lambda _{E})}{(1+\eta \lambda _{E})(\hat{\lambda }_{C}\hat{c}_C^R-\beta {U_R})+\hat{\mu }\hat{c}_C^R}, \end{aligned}$$

60 $$\begin{aligned} \bar{\check{R}}&= \dfrac{(1+\eta \lambda _{E})(\beta {U_R}F\hat{c}_C^R-\hat{\lambda }_{C}\hat{c}_C^R\hat{c}_R)-\hat{c}_C^R\hat{c}_R\hat{\mu }}{\beta (\hat{c}_R-F\hat{c}_C^R)(1+\eta \lambda _{E})}, \end{aligned}$$

61 $$\begin{aligned} \bar{\tilde{A}}&= \dfrac{\hat{c}\hat{c}_C^R}{\lambda _A}\left( \dfrac{(1+\eta \lambda _E)(\hat{c}_R\lambda _R-\beta {U_R}F)+\hat{\mu }\hat{c}_C^R}{(1+\eta \lambda _E)(\hat{c}_R\hat{c}_C^R\hat{\lambda }_C+U_A\beta (F\hat{c}_C^R-\hat{c}_R)-U_R\beta {F}\hat{c}_C^R)+\hat{\mu }\hat{c}_R\hat{c}_C^R}\right) .\nonumber \\ \end{aligned}$$

$\hat{c}_C^R$ has a direct influence on $A$ at leading order on this timescale $(\tau =O(1/\epsilon ))$ meaning that TcdC must remain in the cells for this length of time for it to have any effect upon toxin levels (even when, given that $F\hat{c}_C^R>\hat{c}_R$, TcdC production is faster than that of TcdR). We note from Fig. 9 that this is beyond the onset of stationary phase.

The above workings make it explicit that the value of $F\hat{c}_C^R-\hat{c}_R$ is crucial for determining the overall behaviour of the PaLoc. TcdC can only be effective in lowering toxin production if it is produced at a faster rate than TcdR $(F\hat{c}_C^R>\hat{c}_R)$. Otherwise the system will settle to a steady state with TcdA present in high levels. If TcdC production is switched off at some stage of cell growth ($F=0$ for some $\tau$) then only Case I can ever arise, and TcdC does not affect toxin levels.

Though providing useful suggestions for experiments already, it would be interesting to extend this study to consider the possibility that the value of $F\hat{c}_C^R-\hat{c}_R$ is not constant throughout growth, but rather is subject to variations. If high-frequency experimental time course data were available to suggest that this were the case, this would provide a fascinating extension to the asymptotic analysis.

### Steady-state approximations

**Fig. 10:**
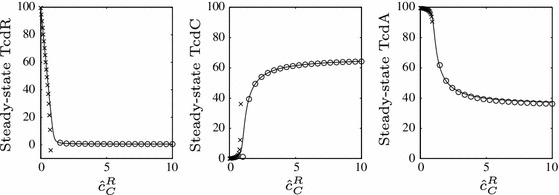
A steady-state plot of TcdR, TcdC and TcdA for varying $\hat{c}_C^R$ with $\epsilon =0.01$. The *solid line* illustrates the numerically-derived steady-state of the full system, while the markers depict our analytical approximations (62)–(75)

Nonlinear systems of the kind presented in this study rarely have explicit steady-state solutions from which information can be gleaned about the influence of specific parameters on the system as a whole. We have already seen in the previous section how the leading-order terms of each equation (these vary depending upon the parameter regime and timescale of interest) can be extracted to obtain tractable asymptotic approximations to the full steady state. These expressions enable clear insight into which parameters, variables and reactions are dominating the behaviour of the full system in the long-term. We extend these approximations here to include lower order terms. We thus summarise our findings of the previous section whilst also gleaning further information about the system, in addition to tracking the steady state of the system in response to variations to the key parameter identified earlier, $\hat{c}_C^R$: the ratio of TcdC to TcdR production—see Fig. 10.

Following the results of Sect. 3.2 which illustrate that TcdC must be present throughout growth for it to affect toxin levels, we here further exploit the aforementioned asymptotic techniques to infer properties of TcdC which would enable it to affect toxin production at steady state if this were indeed the case (i.e., $F=1$ for all $\tau$). For this we require expressions for $C$, $R$ and $A$ (toxin levels follow directly from intracellular TcdA levels and we have seen from Sect. 3.2 that $E$ and $N$ are given at steady state by the appropriate expressions in (47) and (48)). We are able to unravel the magnitude of TcdC’s contribution to the steady-state of the system as a whole in the regimes outlined in the previous section.

Taking $c_C^R=O(1/\epsilon )$ as before (and $c_R=O(1/\epsilon )$ to be fixed), we must consider two regimes: $\hat{c}_C^R<1$ and $\hat{c}_C^R>1$ (we omit $\hat{c}_C^R=1$ for brevity: the leading-order steady states can be obtained from Sect. 3.2.3). In both regimes, we derive the asymptotic expansions of the variables to second order to gain a full picture of the effect of varying $\hat{c}_C^R$ on the system and to glean an estimate of the size of TcdC’s contribution on toxin levels.

#### Regime 1: $\hat{c}_C^R<1$

When $c_C^R=\dfrac{1}{\epsilon }\hat{c}_C^R$ and $\hat{c}_C^R<1$ both TcdC and TcdR production rates are fast but that of TcdC is lower (similar to Case I in Sect. 3.2). The following scalings and approximations hold (bars denote variables at steady state):

62 $$\begin{aligned} \bar{R}=\dfrac{1}{\epsilon }r_{-1}+r_0,\quad \bar{C}=c_0+\epsilon {c_1},\quad \bar{A}=\dfrac{1}{\epsilon }a_{-1}+a_0 \end{aligned}$$

where

63 $$\begin{aligned} r_{-1}&= \dfrac{\hat{c}_R-F\hat{c}_C^R}{\lambda _{R}}, \end{aligned}$$

64 $$\begin{aligned} r_0&= \dfrac{1}{\lambda _{R}(\hat{c}_R-F\hat{c}_C^R)^2}\left( \hat{c}_R(1-U_R\lambda _{R})-F\hat{c}_C^R-\dfrac{{\hat{c}_C^{R2}}F\lambda _{R}}{\beta }\left( \hat{\lambda }_{C}+\dfrac{\hat{\mu }}{2}\right) \right) -\dfrac{\hat{\mu }}{2{\lambda _{R}}^2},\nonumber \\ \end{aligned}$$

65 $$\begin{aligned} c_0&= \dfrac{F\hat{c}_C^R\lambda _{R}}{\beta (\hat{c}_R-F\hat{c}_C^R)}, \end{aligned}$$

66 $$\begin{aligned} c_1&= \dfrac{{\hat{c}_C^{R2}}{\lambda _{R}}^2F}{\beta (\hat{c}_R-F\hat{c}_C^R)^2}\left( \dfrac{\hat{\lambda }_{C}}{\beta }-r_0-\dfrac{\hat{\mu }}{2\beta }\right) , \end{aligned}$$

67 $$\begin{aligned} a_{-1}&= \dfrac{\hat{c}}{\lambda _{A}}, \end{aligned}$$

68 $$\begin{aligned} a_0&= \dfrac{1}{\lambda _{A}}\left( 1-\dfrac{\hat{c}U_A\lambda _{R}}{\hat{c}_R-F\hat{c}_C^R}-\dfrac{\hat{c}\hat{\mu }}{2\lambda _{A}}\right) , \end{aligned}$$

(notice that the leading-order terms, $r_{-1}, c_0$ and $a_{-1}$, concur with (35) and (37) from the dynamic solutions of Sect. 3.2). These approximations are marked by crosses in Fig. 10.

For each variable, the scaling of the leading-order terms with respect to $\epsilon$ reflects its magnitude. Hence in this parameter range, levels of TcdR and TcdA are significantly higher than those of TcdC. Significantly larger amounts of TcdR over TcdC mean that the former has more influence over TcdA levels, preventing TcdC from exerting a negative influence and resulting in high levels of TcdA. Crucially, $\hat{c}_C^R$ only appears in the approximation for $A$ at second order, thus rendering any effect TcdC has to be small relative to that of $R$ via the parameter $\hat{c}$ (the rate of TcdA production).

#### Regime 2: $\hat{c}_C^R>1$

In this scenario TcdC is produced in higher amounts than TcdR (corresponding with Case III of Sect. 3.2) and hence their orders of magnitude switch around and TcdC is able to exert an influence over TcdA. The scalings and approximations are given by

69 $$\begin{aligned} \bar{R}=r_0+\epsilon {r_1},\quad \bar{C}=\dfrac{1}{\epsilon }c_{-1}+c_0,\quad \bar{A}=\dfrac{1}{\epsilon }a_{-1}+a_0, \end{aligned}$$

where

70 $$\begin{aligned} r_0&= \dfrac{(1+\eta \lambda _{E})(\beta {U_R}F\hat{c}_C^R-\hat{\lambda }_{C}\hat{c}_C^R\hat{c}_R)-\hat{c}_C^R\hat{c}_R\hat{\mu }}{\beta (\hat{c}_R-F\hat{c}_C^R)(1+\eta \lambda _{E})}, \end{aligned}$$

71 $$\begin{aligned} r_1&= \dfrac{(r_0+U_R)(r_0\lambda _{R}-1)(\hat{c}_C^R\hat{\mu }/(1+\eta \lambda _{E})-(\hat{\lambda }_{C}\hat{c}_C^R+\beta {r_0}))}{\beta ^2c_{-1}r_0(r_0+U_R)+(\hat{c}_R-\beta {c_{-1}}(2r_0+U_R))(\hat{\mu }\hat{c}_C^R/(1+\eta \lambda _{E})-\hat{\lambda }_{C}\hat{c}_C^R-\beta {r_0})},\nonumber \\ \end{aligned}$$

72 $$\begin{aligned} c_{-1}&= \dfrac{(F\hat{c}_C^R-\hat{c}_R)(1+\eta \lambda _{E})}{(1+\eta \lambda _{E})(\hat{\lambda }_{C}\hat{c}_C^R-\beta {U_R})+\hat{\mu }\hat{c}_C^R}, \end{aligned}$$

73 $$\begin{aligned} c_0&= -\dfrac{(1+\eta \lambda _{E})\beta {c_{-1}}r_1}{\hat{c}_C^R\hat{\mu }+\hat{\lambda }_{C}\hat{c}_C^R+\beta {r_0}}, \end{aligned}$$

74 $$\begin{aligned} a_{-1}&= \dfrac{\hat{c}r_0}{\lambda _{A}(r_0+U_A)}, \end{aligned}$$

75 $$\begin{aligned} a_0&= \dfrac{(1+\eta \lambda _{E})((r_0+U_A)-r_{1}a_{-1}\lambda _{A})-(r0+U_A)\hat{\mu }a_{-1}}{(1+\eta \lambda _{E})(r_0+U_A)\lambda _{A}}. \end{aligned}$$

(69)–(75) are depicted in Fig. 10 with circles; it is evident that these track very closely the true steady state of the full system. In this instance, TcdC levels are notably higher than those of TcdR, and although it is still $O(1/\epsilon )$, TcdA is now affected by $\hat{c}_C^R$ at leading order (notice the dependence of $r_0$ on $\hat{c}_C^R$, and consequently of $a_{-1}$). With an increase to $\hat{c}_C^R$ rather than $\beta$ (as investigated in Sect. 3.1 and Figs. 2, 3 where TcdA levels quickly become negligible), TcdA levels can be lowered but do remain at significant levels in the cell, thus a negative regulator acting via binding to TcdR and produced in much higher amounts than TcdR could feasibly be responsible for the difference between toxin levels in so-called hypervirulent and non-hypervirulent strains (we stress again, however, that the negative regulator must be present *throughout* growth).

Since TcdA remains at $O(1/\epsilon )$, somehow inducing *tcdC* transcription would not be successful in eliminating toxin production, even if TcdC is indeed a negative regulator. An implication being that this approach is unlikely to be a viable option for a novel therapeutic strategy.

In summary, for $\hat{c}_C^R$ to influence TcdA levels at leading order, we see that $\hat{c}_C^R$ must be greater than one. Remembering that we have already scaled $c_C^R$ to be $O(1/\epsilon )$, this amounts to the unscaled nondimensional $c_C^R$ being at least $O(1/\epsilon )$. Translating this into dimensional parameters, we have that

76 $$\begin{aligned} \dfrac{c_C}{c_R^l}\ge {O(1/\epsilon )}. \end{aligned}$$

From our original parameter choice, we also have

77 $$\begin{aligned} c_R=\dfrac{c_R^h}{c_R^l}=O(1/\epsilon ). \end{aligned}$$

Taking (76) and (77) together, it is evident that the rate of TcdC production $(c_C)$ must be at least as high as the highest rate of TcdR production $(c_R^h)$ for it to have a noticeable negative effect upon toxin levels at steady state. This could be investigated experimentally in order to identify if TcdC is produced in sufficient quantities to exert its proposed negative effect. Likewise, this property could be applied to any other protein that is identified as a possible candidate for inhibiting toxin production in this manner: it must be produced at a faster rate than TcdR.

## Discussion

Different experimental approaches have yielded conflicting results regarding the role of TcdC in the PaLoc. In this complementary theoretical study, we have shown how a mathematical model can assist in unravelling the purpose of particular proteins in such networks. While in silico conclusions cannot be assumed automatically to translate into in vivo knowledge, they can be used to recommend experiments for corroboration or simply to provide guidance on the optimal way to obtain more information experimentally. We have indicated that TcdC production should be faster than that of TcdR for it to be able to inhibit TcdR action effectively (if it acts via binding), in addition to the duration of time over which TcdC must actually be produced for it to have a long-term effect (longer than the onset of stationary phase). Both of these things can be investigated experimentally to determine whether TcdC really is a potential candidate for toxin inhibition. These results are equally applicable to alternative proteins, and thus could be used as a generic indicator of potential candidates for toxin inhibition via TcdR-binding.

Current experimental results suggest that TcdC is produced only in early growth (Hundsberger et al. 1997; Govind et al. 2006; Carter et al. 2011) in a laboratory setting (i.e. in batch culture) but it should be considered a possibility that an in vivo infection could consist of cells entirely in exponential growth and thus TcdC could be produced throughout infection. However, this cannot explain the discrepancy in experimental results which suggest that when TcdC is produced only in early growth, it can have a *long-term* effect on toxin levels. Our findings imply that if TcdC does block TcdR action, then toxin production should only be delayed in this setting, agreeing therefore with Bakker et al. (2012) and Cartman et al. (2012) that TcdC is unlikely to be responsible for lowered toxin levels.

The range of $\beta$ (the rate of TcdC binding to TcdR) which allows for a potentially realistic response to TcdC if it were a negative regulator of toxin production (i.e. some inhibition, but not a complete block) is fairly narrow under our parameter regime, suggesting that this would be a very sensitive mechanism (and perhaps therefore somewhat risky if a total switch is not what is required) for *C. difficile* to employ. However, any complete block could be caused by our assumption that, once bound, TcdC and TcdR do not separate (in doing so we were assuming TcdC operates at maximum efficiency, thus if it cannot lower toxin production in the long-term under this assumption we can be more certain it cannot do this under more slack conditions). Importantly, this result could mean that a novel drug which acts by binding TcdR and consequently blocking its action could be effective in quenching *C. difficile* infection via molecular titration. Overexpressing production of a negative regulator, whether it acts by binding the protein or blocking *tcdR* transcription, appears to be a less promising strategy since in our simulations toxin production was not abolished in these cases.

Of course, a number of additional assumptions were made in the formulation of the model, but we stress that these do not affect our results. For instance, TcdE may not actually be responsible for toxin secretion and therefore the formation of extracellular toxins (as argued by Olling et al. 2012), but all of our conclusions regarding toxin levels can be drawn from the variables representing intracellular TcdA (extracellular quantities were really only considered in Sect. 3.1 for completeness). Similarly, including TcdB in the model would have given identical qualitative results to those seen for TcdA.

This study highlights the benefits of using a combined numerical and asymptotic approach to investigate gene regulation networks. While both enable a visualisation of the dynamics of the system, including investigating a range of parameter values, the asymptotic analysis permits significant insight into which reactions, variables and parameters are actually determining the behaviour of the system. The generation of smaller and less complicated subsystems which capture the behaviour of the full system on timescales or parameter regimes of interest are useful not only to improve our understanding of the system as a whole and to simplify the underlying mathematics, but also to pinpoint elements worthy of further experimental investigation.

## Summary

If elements of the pathogenesis-related regulatory network of *C. difficile* are to be used as targets of novel drugs (in order to minimise the likelihood of the bacteria developing resistance to the drugs), it is crucial that efforts across a range of disciplines be adopted to ensure we fully understand the implications of interfering with the life-cycle of this important bacterium.

TcdC has long been assumed to play a pivotal role in toxin production with only more recent studies suggesting that its contribution is in fact negligible. Our findings here support the hypothesis that TcdC negatively regulates toxin production in conventional (i.e. non-hypervirulent) strains and that a lack of its functionality could be responsible for any increase in toxins in hypervirulent strains *only* if it is produced throughout growth and in higher quantities than TcdR. We thus present model-derived properties which can be investigated in a laboratory environment to lend support to either of the biological hypotheses. This will yield experimental data which can further inform the model and strengthen the reliability of future predictions.

## Appendix A: Time-dependent asymptotic analysis

We here provide the mathematical detail behind the asymptotic analysis on the system below, as outlined in Sect. 3.2.

78 $$\begin{aligned} \dfrac{dN}{d\tau }&= N(1-N)-\epsilon \hat{\mu }\left( \dfrac{E}{E+\eta }\right) N, \ \end{aligned}$$

79 $$\begin{aligned} \dfrac{dE}{d\tau }&= 1-\lambda _{E}E-E(1-N), \end{aligned}$$

80 $$\begin{aligned} \dfrac{dA}{d\tau }&= 1+\dfrac{1}{\epsilon }\dfrac{\hat{c}R}{(R+U_A)}-\lambda _{A}A-A(1-N), \end{aligned}$$

81 $$\begin{aligned} \dfrac{dR}{d\tau }&= 1+\dfrac{1}{\epsilon }\dfrac{\hat{c}_RR}{(R+U_R)}-\beta {C}R-\lambda _{R}R-R(1-N), \end{aligned}$$

82 $$\begin{aligned} \dfrac{dC}{d\tau }&= F-\epsilon \dfrac{\beta }{\hat{c}_C^R}CR-\epsilon {\hat{\lambda }_{C}}C-C(1-N). \end{aligned}$$

### A.1 Timescale 1: $\tau =\epsilon \hat{\tau }$

On this first timescale, all variables except $\tau$ are $O(1)$. The scaling $\tau =\epsilon \hat{\tau }$ enables the appearance of the fastest reactions to appear here at leading order. We assume all initial conditions are $O(1)$ implying that the bacteria do not produce toxins in significant quantities initially and do not require scaling. The system (78)–(82) becomes

83 $$\begin{aligned} \dfrac{dN}{d\hat{\tau }}&= \epsilon {N}(1-N)-\epsilon ^2\hat{\mu } \left( \dfrac{E}{E+\eta }\right) N, \end{aligned}$$

84 $$\begin{aligned} \dfrac{dE}{d\hat{\tau }}&= \epsilon -\epsilon \lambda _{E}E-\epsilon {E}(1-N), \end{aligned}$$

85 $$\begin{aligned} \dfrac{dA}{d\hat{\tau }}&= \epsilon +\dfrac{\hat{c}R}{R+U_A}-\epsilon \lambda _{A}A-\epsilon {A}(1-N), \end{aligned}$$

86 $$\begin{aligned} \dfrac{dR}{d\hat{\tau }}&= \epsilon +\dfrac{\hat{c}_RR}{R+U_R}-\epsilon \beta {C}R-\epsilon \lambda _{R}R-\epsilon {R}(1-N), \end{aligned}$$

87 $$\begin{aligned} \dfrac{dC}{d\hat{\tau }}&= \epsilon {F}-\epsilon ^2\hat{\lambda }_{C}C-\epsilon ^2\dfrac{\beta }{\hat{c}_C^R}CR-\epsilon {C}(1-N), \end{aligned}$$

meaning that the $O(1)$ terms yield the following leading-order behaviour:

88 $$\begin{aligned}&N = N(0), \qquad E=E(0), \qquad C=C(0), \end{aligned}$$

89 $$\begin{aligned}&R+U_R\ln (R)=\hat{c}_R\hat{\tau }+R(0)+U_R\ln (R(0)), \end{aligned}$$

90 $$\begin{aligned}&A = \int \dfrac{\hat{c}R}{R+U_A}\,d\hat{\tau }. \end{aligned}$$

In order to link to the following timescale we must derive the long-term behaviour of each variable and match these to the subsequent short-term dynamics: we have that

91 $$\begin{aligned} R\sim \hat{c}_R\hat{\tau } \quad \hbox {and} \quad A\sim \hat{c}\hat{\tau } \quad \hbox {for large} \quad \hat{\tau } \end{aligned}$$

on this timescale, while the other variables remain constant. This informs our choice of variable scalings on the subsequent timescale: since both $R$ and $A$ behave like $\hat{\tau }$ for large $\hat{\tau }$, they must be scaled with the same power of $\epsilon$ as is $\hat{\tau }$ to move to the second timescale. The relevant power of $\epsilon$ is chosen to bring the mathematically appropriate terms into the leading-order behaviour on the second timescale.

### A.2 Timescale 2: $\hat{\tau }=\epsilon ^{-1}\tilde{\tau }$, $A=\epsilon ^{-1}\tilde{A}$, $R=\epsilon ^{-1}\tilde{R}$

When identifying the scalings required to move to a next timescale, it is helpful to examine the comparisons between the approximations on the previous timescale and the full system (Figs. 7, 8, 9). It is clear that the approximations for $A$ and $R$ grow too fast on the first timescale. Similarly, those for $N$, $E$ and $C$ do not grow at all. These facilitate the derivation of the above scalings for the second timescale. The rescaled problem reads

92 $$\begin{aligned} \dfrac{dN}{d\tilde{\tau }}&= N(1-N)-\epsilon \hat{\mu } \left( \dfrac{E}{E+\eta }\right) N, \end{aligned}$$

93 $$\begin{aligned} \dfrac{dE}{d\tilde{\tau }}&= 1-\lambda _{E}E-E(1-N), \end{aligned}$$

94 $$\begin{aligned} \dfrac{d\tilde{A}}{d\tilde{\tau }}&= \epsilon +\dfrac{\hat{c}\tilde{R}}{\tilde{R}+\epsilon {U_A}}-\lambda _{A}\tilde{A}-\tilde{A}(1-N), \end{aligned}$$

95 $$\begin{aligned} \dfrac{d\tilde{R}}{d\tilde{\tau }}&= \epsilon +\dfrac{\hat{c}_R\tilde{R}}{\tilde{R}+\epsilon {U_R}}-\beta {C}\tilde{R}-\lambda _{R}\tilde{R}-\tilde{R}(1-N), \end{aligned}$$

96 $$\begin{aligned} \dfrac{dC}{d\tilde{\tau }}&= F-\epsilon \hat{\lambda }_{C}C-\dfrac{\beta }{\hat{c}_C^R}C\tilde{R}-C(1-N). \end{aligned}$$

Notice that the equations for $N$, $E$ and $A$ decouple from the remaining system at leading order and can be solved to give

97 $$\begin{aligned} N&= \dfrac{N(0)e^{\tilde{\tau }}}{1+N(0)(e^{\tilde{\tau }}-1)}, \end{aligned}$$

98 $$\begin{aligned} E&= \left( \dfrac{1-N(0)}{e^{\tilde{\tau }(\lambda _{E}+1)}}+\dfrac{N(0)}{e^{\lambda _{E}\tilde{\tau }}}\right) \int \dfrac{e^{\tilde{\tau }(\lambda _{E}+1)}}{1-N(0)+N(0)e^{\tilde{\tau }}}\,d\tilde{\tau }, \end{aligned}$$

99 $$\begin{aligned} \tilde{A}&= \left( \dfrac{1-N(0)}{e^{\tilde{\tau }(\lambda _{A}+1)}}+\dfrac{N(0)}{e^{\lambda _{A}\tilde{\tau }}}\right) \int \dfrac{\hat{c}e^{\tilde{\tau }(\lambda _{A}+1)}}{1-N(0)+N(0)e^{\tilde{\tau }}}\,d\tilde{\tau }, \end{aligned}$$

where

100 $$\begin{aligned} N\sim {1}, \qquad E\sim \dfrac{1}{\lambda _{E}},\qquad \tilde{A}\sim \dfrac{\hat{c}}{\lambda _{A}} \end{aligned}$$

for large $$\tilde{\tau }$$.

Adopting (100) and combining the leading-order terms of (95) and (96) enables us to obtain the large $\tilde{\tau }$ behaviour of $C$ and $\tilde{R}$:

101 $$\begin{aligned} \dfrac{d}{d\tilde{\tau }}(\hat{c}_C^RC-\tilde{R})&\sim (F\hat{c}_C^R-\hat{c}_R)+\lambda _{R}\tilde{R}. \end{aligned}$$

The dynamics of these two variables are determined by whether $F\hat{c}_C^R-\hat{c}_R$ is negative, zero or positive. We examine each case separately (note that (100) holds for all three scenarios outlined below).

#### A.2.1 Case I: $F\hat{c}_C^R< \hat{c}_R$

If $F\hat{c}_C^R< \hat{c}_R$, the leading-order system evolves to steady state on this second timescale, namely

102 $$\begin{aligned} \bar{R}\sim \dfrac{\hat{c}_R-F\hat{c}_C^R}{\lambda _{R}}, \qquad \bar{C}\sim \dfrac{F\hat{c}_C^R\lambda _{R}}{\beta (\hat{c}_R-F\hat{c}_C^R)}, \end{aligned}$$

(see Fig. 7) and therefore no further timescales are required for this parameter regime.

#### A.2.2 Case II: $F\hat{c}_C^R=\hat{c}_R$

Examination of the numerical solutions illustrates that in (101) $d\tilde{R}/d\tilde{\tau }$ is negligible in relation to the other terms. Thus,

103 $$\begin{aligned} \tilde{R}\sim \dfrac{\hat{c}_C^R}{\lambda _{R}}\dfrac{dC}{d\tilde{\tau }}. \end{aligned}$$

Substituting (103) back into the leading order terms of (96) (again, using $N\sim {1}$) gives for large $\tilde{\tau }$

104 $$\begin{aligned} \left( 1+\dfrac{\beta }{\lambda _{R}}C\right) \dfrac{dC}{d\tilde{\tau }}&\sim {F}. \end{aligned}$$

However, because $$C\gg 1 $$,

105 $$\begin{aligned} C\sim \left( \dfrac{2F\lambda _{R}}{\beta }\tilde{\tau }\right) ^{\frac{1}{2}}. \end{aligned}$$

From (103) this gives

106 $$\begin{aligned} \tilde{R}\sim \dfrac{F\hat{c}_C^R}{\beta }\left( \dfrac{2F\lambda _{R}}{\beta }\tilde{\tau }\right) ^{-\frac{1}{2}}. \end{aligned}$$

#### A.2.3 Case III: $F\hat{c}_C^R>\hat{c}_R$

Examination of the numerical solution to the equations shows that both $d\tilde{R}/d\tilde{\tau }$ and $\lambda _{R}\tilde{R}$ become negligible in relation to the other terms in (101) under this parameter regime. Thus

107 $$\begin{aligned} \dfrac{dC}{d\tilde{\tau }}\sim \dfrac{F\hat{c}_C^R-\hat{c}_R}{\hat{c}_C^R} \end{aligned}$$

which gives

108 $$\begin{aligned} C\sim \left( \dfrac{F\hat{c}_C^R-\hat{c}_R}{\hat{c}_C^R}\right) \tilde{\tau }, \end{aligned}$$

and hence from (95) and (100) (remembering that the contributions of $d\tilde{R}/d\tilde{\tau }$ and $\lambda _{R}\tilde{R}$ are negligible in relation to other terms in (95) also)

109 $$\begin{aligned} \tilde{R}\sim \dfrac{\hat{c}_R\hat{c}_C^R}{\beta (F\hat{c}_C^R-\hat{c}_R)\tilde{\tau }} \end{aligned}$$

for large $$\tilde{\tau }$$.

### A.3 Timescale 3

#### A.3.1 Case I: $F\hat{c}_C^R< \hat{c}_R$

When $F\hat{c}_C^R< \hat{c}_R$, the system has already evolved to steady state on Timescale 2 and therefore no further discussion is needed here.

#### A.3.2 Case II: $F\hat{c}_C^R=\hat{c}_R$, $\tilde{\tau }=\epsilon ^{-1}\check{\tau }$, $C=\epsilon ^{-\frac{1}{2}}\check{C}$, $\tilde{R}=\epsilon ^{\frac{1}{2}}\check{R}$

On the previous timescale

$$\begin{aligned} {C}\sim \tilde{\tau }^{\frac{1}{2}} \quad \hbox {and} \quad R\sim \tilde{\tau }^{-\frac{1}{2}} \end{aligned}$$

(see (105) and (106)). Thus, if $\tilde{\tau }$ is scaled with $\epsilon ^\alpha$ then $C$ must be scaled with $\epsilon ^{\frac{\alpha }{2}}$ and $R$ with $\epsilon ^{-\frac{\alpha }{2}}$. The above scalings (that set $\alpha =-1$ to achieve a new balance by bringing to leading-order TcdR production and TcdC loss–see Fig. 8) abide to this rule. On this third timescale, therefore, if the rates of TcdC and TcdR production are identical, we have

110 $$\begin{aligned} \epsilon \dfrac{dN}{d\check{\tau }}&= N(1-N)-\epsilon \hat{\mu }\left( \dfrac{E}{E+\eta }\right) N, \end{aligned}$$

111 $$\begin{aligned} \epsilon \dfrac{dE}{d\check{\tau }}&= 1-\lambda _{E}E-E(1-N), \end{aligned}$$

112 $$\begin{aligned} \epsilon \dfrac{dA}{d\check{\tau }}&= \epsilon +\dfrac{\hat{c}\check{R}}{\check{R}+\epsilon ^{\frac{1}{2}}U_A}-\lambda _{A}A-A(1-N), \end{aligned}$$

113 $$\begin{aligned} \epsilon ^{\frac{3}{2}}\dfrac{d\check{R}}{d\check{\tau }}&= \epsilon +\dfrac{\hat{c}_R\check{R}}{\check{R}+\epsilon ^{\frac{1}{2}}U_R}-\beta {\check{C}}{\check{R}}-\epsilon ^{\frac{1}{2}}\lambda _{R}\check{R}-\epsilon ^{\frac{1}{2}}{\check{R}}(1-N), \end{aligned}$$

114 $$\begin{aligned} \epsilon \dfrac{d\check{C}}{d\check{\tau }}&= \epsilon ^{\frac{1}{2}}F-\epsilon \hat{\lambda }_{C}\check{C}-\epsilon ^{\frac{1}{2}}\dfrac{\beta }{\hat{c}_C^R}\check{C}\check{R}-\check{C}(1-N). \end{aligned}$$

The first three of these equations give

115 $$\begin{aligned} N\sim 1-\epsilon \hat{\mu }\left( \dfrac{1}{1+\eta \lambda _{E}}\right) , \end{aligned}$$

(the $O(\epsilon )$ term being needed to obtain the leading-order balance in (114)),

116 $$\begin{aligned} E\sim \dfrac{1}{\lambda _{E}}, \qquad A\sim \dfrac{\hat{c}}{\lambda _{A}}. \end{aligned}$$

Combining (113) and (114) and taking leading-order terms, we obtain the following expression

117 $$\begin{aligned} \hat{c}_C^R\dfrac{d\check{C}}{d\check{\tau }}&\sim \lambda _{R}\check{R}+\dfrac{\hat{c}_RU_R}{\check{R}}-\left( \hat{\lambda }_{C}+\dfrac{\hat{\mu }}{1+\eta \lambda _{E}}\right) \hat{c}_C^R\check{C}, \end{aligned}$$

but from (114) and the condition that $F\hat{c}_C^R=\hat{c}_R$,

118 $$\begin{aligned} \check{R}\sim \dfrac{\hat{c}_R}{\beta \check{C}}. \end{aligned}$$

Hence,

119 $$\begin{aligned} \dfrac{d\check{C}}{d\hat{\tau }}\sim \dfrac{F\lambda _{R}}{\beta \check{C}}-\left( {\hat{\lambda }_{C}}+\dfrac{\hat{\mu }}{1+\eta \lambda _{E}}-\dfrac{FU_R\beta }{\hat{c}_R}\right) \check{C}, \end{aligned}$$

with $\check{C}\rightarrow {0}$ as $\check{\tau }\rightarrow {0}$. This can be solved to give

120 $$\begin{aligned} \check{C}&\sim \dfrac{\sqrt{\rho \theta (1-e^{-2\rho \check{\tau }})}}{\rho }, \end{aligned}$$

121 $$\begin{aligned} \check{R}&\sim \dfrac{\hat{c}_R\rho \sqrt{\rho \theta (1-e^{-2\rho \check{\tau }})}}{\beta \rho \theta (1-e^{-2\rho \check{\tau }})}, \end{aligned}$$

where

122 $$\begin{aligned} \rho =\hat{\lambda }_C+\dfrac{\hat{\mu }}{1+\eta \lambda _E}-\dfrac{FU_R\beta }{\hat{c}_R} \quad \hbox {and} \quad \theta =\dfrac{F\lambda _R}{\beta }. \end{aligned}$$

The resulting approximations are depicted in Fig. 8.

#### A.3.3 Case III: $F\hat{c}_C^R>\hat{c}_R$, $\tilde{\tau }=\epsilon ^{-1}\check{\tau }$, $C=\epsilon ^{-1}\check{C}$, $\tilde{R}=\epsilon \check{R}$

On the previous timescale

$$\begin{aligned} C\sim \tilde{\tau } \quad \hbox {and} \quad \tilde{R}\sim \tilde{\tau }^{-1} \end{aligned}$$

for this Case (see (108) and (109)). Therefore, (following the reasoning outlined for Case II) the above scalings facilitate the transition between the two timescales. In this case, where TcdC production occurs at a faster rate than that of TcdR, we have on this final timescale:

123 $$\begin{aligned} \epsilon \dfrac{dN}{d\check{\tau }}&= N(1-N)-\epsilon \hat{\mu }\left( \dfrac{E}{E+\eta }\right) N, \end{aligned}$$

124 $$\begin{aligned} \epsilon \dfrac{dE}{d\check{\tau }}&= 1-\lambda _{E}E-E(1-N), \end{aligned}$$

125 $$\begin{aligned} \epsilon \dfrac{d\tilde{A}}{d\check{\tau }}&= \epsilon +\dfrac{\hat{c}\check{R}}{\check{R}+U_A}-\lambda _{A}\tilde{A}-\tilde{A}(1-N), \end{aligned}$$

126 $$\begin{aligned} \epsilon ^2\dfrac{d\check{R}}{d\check{\tau }}&= \epsilon +\dfrac{\hat{c}_R\check{R}}{\check{R}+U_R}-\beta {\check{C}}{\check{R}}-\epsilon \lambda _{R}\check{R}-\epsilon {\check{R}}(1-N), \end{aligned}$$

127 $$\begin{aligned} \epsilon \dfrac{d\check{C}}{d\check{\tau }}&= \epsilon {F}-\epsilon \hat{\lambda }_{C}\check{C}-\epsilon \dfrac{\beta }{\hat{c}_C^R}\check{C}\check{R}-\check{C}(1-N), \end{aligned}$$

so that (115) holds also in this case. The remaining approximations are given by

128 $$\begin{aligned}&E \sim \dfrac{1}{\lambda _{E}}, \quad \check{R}\sim \dfrac{\hat{c}_R}{\beta {\check{C}}}-U_R,\quad \tilde{A}\sim \dfrac{\hat{c}}{\lambda _{A}}\left( \dfrac{\hat{c}_R-U_R\beta \check{C}}{\hat{c}_R+(U_A-U_R)\beta \check{C}}\right) , \end{aligned}$$

129 $$\begin{aligned}&\dfrac{d\check{C}}{d\check{\tau }} = F-\dfrac{\hat{c}_R}{\hat{c}_C^R}+ \left( \dfrac{\beta {U_R}}{\hat{c}_C^R}-\hat{\lambda }_{C}-\dfrac{\hat{\mu }}{1+\eta \lambda _{E}}\right) \check{C}. \end{aligned}$$

(129) can be solved to give

130 $$\begin{aligned} \check{C}\sim \dfrac{(F\hat{c}_C^R-\hat{c}_R)(1+\eta \lambda _{E})}{(1+\eta \lambda _{E})(\beta {U_R}-\hat{\lambda }_C\hat{c}_C^R)-\hat{\mu }\hat{c}_C^R}\left( \exp \left( \left( \frac{\beta {U_R}}{\hat{c}_C^R}-\hat{\lambda }_{C}-\frac{\hat{\mu }}{1+\eta \lambda _{E}}\right) \check{\tau }\right) -1\right) \!, \end{aligned}$$

(matching to $\check{C}\sim ((F\hat{c}_C^R-\hat{c}_R)/\hat{c}_C^R)\check{\tau }$ as $\check{\tau }\rightarrow {0}$). The solutions for $\check{R}$ and $A$ follow.

At steady-state therefore we have

131 $$\begin{aligned} \bar{\check{C}}&= \dfrac{(F\hat{c}_C^R-\hat{c}_R)(1+\eta \lambda _{E})}{(1+\eta \lambda _{E})(\hat{\lambda }_{C}\hat{c}_C^R-\beta {U_R})+\hat{\mu }\hat{c}_C^R}, \end{aligned}$$

132 $$\begin{aligned} \bar{\check{R}}&= \dfrac{(1+\eta \lambda _{E})(\beta {U_R}F\hat{c}_C^R-\hat{\lambda }_{C}\hat{c}_C^R\hat{c}_R)-\hat{c}_C^R\hat{c}_R\hat{\mu }}{\beta (\hat{c}_R-F\hat{c}_C^R)(1+\eta \lambda _{E})}, \end{aligned}$$

133 $$\begin{aligned} \bar{\tilde{A}}&= \dfrac{\hat{c}\hat{c}_C^R}{\lambda _A}\left( \dfrac{(1+\eta \lambda _E)(\hat{c}_R\lambda _R-\beta {U_R}F)+\hat{\mu }\hat{c}_C^R}{(1+\eta \lambda _E)(\hat{c}_R\hat{c}_C^R\hat{\lambda }_C+U_A\beta (F\hat{c}_C^R-\hat{c}_R)-U_R\beta {F}\hat{c}_C^R)+\hat{\mu }\hat{c}_R\hat{c}_C^R}\right) .\nonumber \\ \end{aligned}$$

## Footnotes

1 A third, the binary toxin CDT (*C. difficile* transferase), plays a much less pronounced role (Carter et al. 2007) and is not known to interact with the proteins in Fig. 1. It is accordingly neglected from the model.
